# Quantitative whole-cell MALDI-TOF MS fingerprints distinguishes human monocyte sub-populations activated by distinct microbial ligands

**DOI:** 10.1186/s12896-015-0140-1

**Published:** 2015-04-11

**Authors:** Damien Portevin, Valentin Pflüger, Patricia Otieno, René Brunisholz, Guido Vogel, Claudia Daubenberger

**Affiliations:** Department of Medical Parasitology and Infection Biology, Swiss TPH, Basel, Switzerland; University of Basel, 4002 Basel, Switzerland; Mabritec AG, Riehen, Switzerland; Functional Genomics Center Zurich, ETH Zurich and University of Zurich, Zurich, Switzerland

**Keywords:** Whole-cell mass spectrometry, MALDIquant, Fingerprints, Monocyte, CD14, CD16, MDC8

## Abstract

**Background:**

Conventionally, human monocyte sub-populations are classified according to surface marker expression into classical (CD14^++^CD16^−^), intermediate (CD14^++^CD16^+^) and non-classical (CD14^+^CD16^++^) lineages. The involvement of non-classical monocytes, also referred to as proinflammatory monocytes, in the pathophysiology of diseases including diabetes mellitus, atherosclerosis or Alzheimer’s disease is well recognized. The development of novel high-throughput methods to capture functional states within the different monocyte lineages at the whole cell proteomic level will enable real time monitoring of disease states.

**Results:**

We isolated and characterized (pan-) monocytes, mostly composed of classical CD16^−^ monocytes, versus autologous CD16^+^ subpopulations from the blood of healthy human donors (n = 8) and compared their inflammatory properties in response to lipopolysaccharides and *M.tuberculosis* antigens by multiplex cytokine profiling. Following resting and in vitro antigenic stimulation, cells were recovered and subjected to whole-cell mass spectrometry analysis. This approach identified the specific presence/absence of m/z peaks and therefore potential biomarkers that can discriminate pan-monocytes from their CD16 counterparts. Furthermore, we found that semi-quantitative data analysis could capture the subtle proteome changes occurring upon microbial stimulation that differentiate resting, from lipopolysaccharides or *M. tuberculosis* stimulated monocytic samples.

**Conclusions:**

Whole-cell mass spectrometry fingerprinting could efficiently distinguish monocytic sub-populations that arose from a same hematopoietic lineage. We also demonstrate for the first time that mass spectrometry signatures can monitor semi-quantitatively specific activation status in response to exogenous stimulation. As such, this approach stands as a fast and efficient method for the applied immunology field to assess the reactivity of potentially any immune cell types that may sustain health or promote related inflammatory diseases.

**Electronic supplementary material:**

The online version of this article (doi:10.1186/s12896-015-0140-1) contains supplementary material, which is available to authorized users.

## Background

Five to 15% of human blood mononuclear cells are monocytes. Upon extravasation into inflamed tissue, their ability to differentiate into monocyte-derived macrophages and dendritic cells make them key components of the innate immune system to initiate adaptive immune responses and contribute to tissue homeostasis upon resolution of inflammation [1]. Based on the most recent nomenclature, monocytes can be divided into classical (CD14^++^CD16^−^), intermediate CD14^++^CD16^+^ and non-classical (CD14^+^CD16^++^) short-lived cells which are constantly replenished from a common myeloid progenitor [2-4]. Their phenotypic characterization is usually performed by flow cytometry using a gating strategy in combination with HLA-DR labelling to exclude the measurement of NK cells and granulocytes expressing the CD16 marker as well [5].

Based on their CD16 expression levels, monocyte subpopulations have different chemotactic, phagocytic and inflammatory abilities [6] and distinct propensities to differentiate into professional antigen presenting cells [7]. In fact, blood monocyte composition is heterogeneous between human individuals and gender [8] and within individuals it can be substantially altered by infections such as septicaemia, HIV and *M. tuberculosis* infection [9-11] during which the CD16^+^ compartment can increase dramatically. In the case of *M. tuberculosis* infection, a functional deficiency in the CD16^+^ subset to recall specific immune responses due to an impaired dendritic cell differentiation has been notably described [12]. In contrast, a human genetic trait associated to an absent CD16+ compartment in the blood was not necessarily associated with disease [13]. Nevertheless, these inherent and induced discrepancies between humans have led to a series of reports describing association of the blood monocyte composition and recruitment with a particular emphasis on CD16+ monocytes in the pathogenesis of non-communicable inflammatory disorders such as atherosclerosis [14], stroke [15], rheumatoid arthritis [16] and diabetes complications [17] but also Alzheimer’s disease [18], and experimental models of human disorder multiple sclerosis [19], tumour genesis and metastasis [20]. In that context, we aimed to develop a method that could assess CD16^+^ monocyte functionalities for the diagnosis/prognosis but also severity or resolution monitoring of those diseases which may be particularly meaningful in the context of immune-based therapeutics targeting the recruitment of monocytes [21].

Untargeted mass spectrometry analysis of complex samples such as entire prokaryotic or eukaryotic cell lysates that are directly spotted in the MALDI ionisation/desorption matrix offers a fast method for rapid identification of bacteria [22] but also the discrimination/authentication of mammalian and insect cell lines [23-26]. This approach notably prevents any potential bias or reproducibility issues due to purification or fractionation steps prior mass spectrometry analysis. Whole-cell MALDI-TOF “fingerprints” or “signatures” performed on human primary blood cells were shown to reproducibly discriminate lymphocytes from monocytes or granulocytes or between macrophage subtypes with relatively low starting material ranging between 25 × 10^2^ to 5 × 10^4^ cells [27,28]. In that context, we aimed to assess whether whole-cell MALDI-TOF fingerprinting would have enough discriminatory power to i) distinguish human monocyte subpopulations and ii) monitor activation profiles of monocytes exposed to distinct microbial ligand. We purified monocytes from healthy individuals (n = 8) and isolated autologous CD16^+^ subpopulations. Cell preparations were immunologically characterized and following stimulation with LPS and *M. tuberculosis* derived antigens subjected to MALDI-TOF analysis. We extracted semi-quantitative mass spectrometry data to highlight monocytic subset discriminatory biomarkers as well as specific microbial activation profiles.

## Methods

### Ethics statement

Fresh blood packs (buffy coat) were purchased anonymously from the Blutspendezentrum SRK beider Basel, Switzerland. In compliance with the Helsinki Declaration, signed informed consents stating specifically that “the donation or certain components thereof be used for medical research after definitive anonymization” was obtained prior blood donation. Consent form was accessed on October 31^st^ 2014 and can be found here: http://blutspende-basel.ch/fileadmin/BSZ/docs/blutspende/2015_Blutspendeaufgebot_en.pdf. An ethics board approval for this study was consequently not required.

### Blood processing and monocyte isolation

Peripheral Blood Mononuclear Cells (PBMCs) were immediately isolated by density centrifugation using pre-filled Greiner Bio-One Leucosep® tubes according to the manufacturer’s recommendations. PBMCs rings were collected and washed twice in PBS before final suspension at 20 × 10^6^ cells/ml in ice-cold freezing medium (50% RPMI-1640, 40% fetal bovine serum, 10% DMSO) and transferred at −80°C in Nalgene® Mr. Frosty for short-term storage (<1 month). For monocyte isolation, 40 × 10^6^ to 60 × 10^6^ cells of cryopreserved PBMCs were thawed and quickly washed with 13 ml ice-cold RPMI-1640. Median cell viability upon recovery was assessed by trypan blue exclusion (88.37%, IQR: 82.1-97). Pan- and CD16+ monocyte purification were performed using Pan Monocyte Isolation Kit and CD16+ Monocyte Isolation Kit respectively according to manufacturer’s instructions (Miltenyi Biotec GmbH).

### Flow cytometry analysis

Prior final washing steps, 100 μl suspension of PBMCs, pan-monocytes and CD16 monocytes were spared and washed with PBS 1% FCS before antibody staining for 15 min on ice with anti-CD14 FITC (MΦP9, Becton Dickinson), anti-CD16-PE (Leu11c, Becton Dickinson) and anti-Slan (MDC8, Miltenyi Biotec). After washing, antibody labelled cell preparation were immediately analysed on a BD FACSCalibur apparatus (BD Biosciences) and data analyzed using FlowJo 9.5.2 (Treestar).

### Cell culture conditions

Monocyte suspensions were cultivated in 96-well flat-bottom tissue culture treated plates at a density of 2 × 10^5^ cells per well in complete medium (RPMI-1640 (Gibco Life Technologies™) complemented with 5% heat-inactivated fetal bovine serum (PAA Laboratories GmbH), 100 mM Na-pyruvate (Gibco), Penicillin/Streptomycin (50 U & 50 μg/ml respectively, Gibco) for 24 hours at 37°C in a humidified atmosphere with 5% CO2 in the presence or absence (resting) of PPD (RT50, Statens Serum Institute, 5 μg/ml final) or LPS (O111:B4, 10 ng/ml final, Sigma-Aldrich). After 24 hours of resting/incubation period, tissue culture plates were placed on ice for 15 minutes and the cells re-suspended by vigorous pipetting, pelleted by centrifugation (250 RCF) and cell pellets transferred at −80°C.

### Cytokine profiling

Cytokine concentrations present in monocyte culture supernatants were measured using Bio-Plex Pro™ Human Cytokine Group I kit according to manufacturer’s instructions and data acquired on a Luminex® 200™ System.

### Whole-cell mass spectrometry

Cryopreserved cell pellets were washed with 70% ethanol and briefly vortexed before centrifugation (10 min, 16000 RCF). Supernatants were discarded, cell pellets dried for 1 minute at room temperature and finally solubilized with 10 μl formic acid 10%, mixed with 2 volumes of a saturated sinapinic acid solution (40 mg for 1 ml of acetonitrile 60%/H20 37%/TFA 3%) and spotted in quadruplicates on a MALDI-TOF chip. External calibration was performed using ribosomal corresponding m/z signals from whole-cell E. Coli (DH5α). Mass spectra (m/z mass range: 3000 to 30000) were acquired on a Shimadzu Biotech Axima Confidence.

### Quantitative analysis of mass spectrometry data using MALDIquant R package

Mass spectra were pre-aligned to conserved m/z peaks at 4078.8 and 14019.1, exported as mzXML files using MALDI-MS Shimadzu Biotech Launchpad 2.8.1 (Kratos Analytical Ltd) and imported using R Studio version 0.98.945 and MALDIquant Foreign R package. Using MALDIquant package [29], mass spectra were square-root transformed, smoothed using Savitz-Golay-Filter, baseline corrected using TopHat method and intensity values median-normalized so as to set to one the median of peak intensities of each mass spectrum individually before alignment using the Lowess warping algorithm. Peak detection was performed using a signal-to-noise ratio of 3. Peak list and their respective intensities were retrieved and peak intensities obtained from each quadruplicates averaged using OpenOfficeCalc before further statistical analysis.

### Statistical analysis

Wilcoxon signed rank tests, two-way ANOVA with Bonferroni post-test corrections were performed and scatter-plots generated using GraphPad prism (version 4.03, San Diego, CA, USA). Principal component analyses using correlation matrix were conducted using Paleontological Statistics Software package for education and data analysis, version 2.17c [30].

## Results and discussion

### Phenotypic and functional characterization of Pan- versus autologous CD16+ monocytes

**Figure 1:**
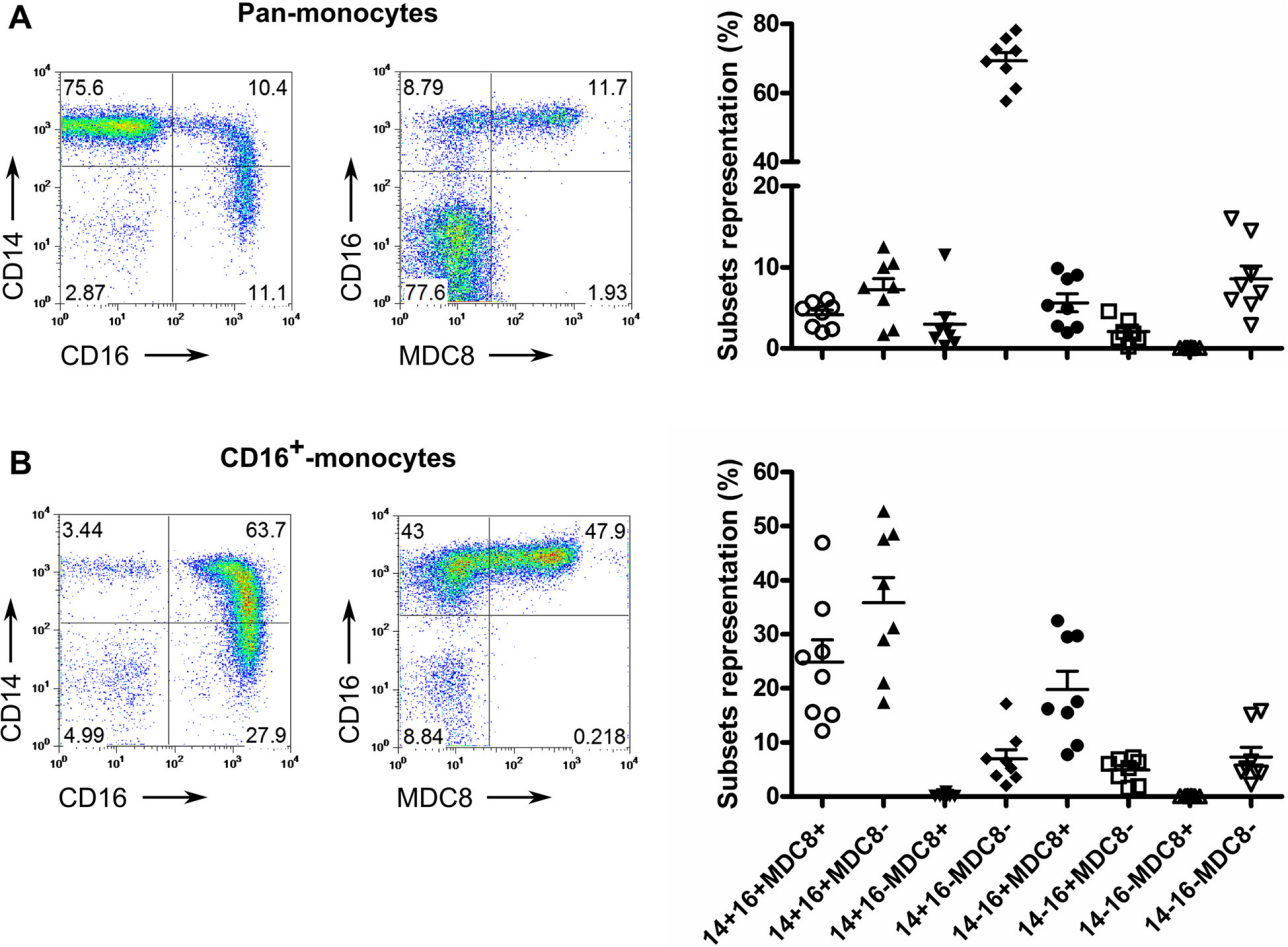
Phenotypic characterization of isolated monocyte populations. Representative FACS-plot and scatter-plot frequencies of **A)** Pan-monocytes and **B)** CD16^+^ monocytes based on the expression of CD14 versus CD16 (left plots) and CD16 versus P-selectin glycoprotein ligand-1 (MDC8 antibody specificity [31], right plots). Scatter-plots summarizing frequencies of each sub-population based on the combinations between the three monocytic markers are shown across all monocytes preparations (n = 8 healthy donors).

Cryo-preserved PBMCs from adult healthy donors (n = 8) were thawed and used to enrich for pan-monocyte and CD16^+^ monocyte sub-populations. Purified cell populations were analysed by flow cytometry using antibodies directed against the LPS receptor (CD14), the Fc-gamma receptor III, CD16 and the P-selectin glycoprotein ligand-1 (Figure 1A). Flow cytometry data were subjected to Boolean gating analysis to evaluate the representation of individual sub-populations expressing one or several of these cell surface markers. The majority of the pan-monocyte cells expressed exclusively CD14 and no CD16 or MDC8 ligand (CD14^+^/CD16^−^/MDC8^−^) (Figure 1B). Enriched CD16^+^ monocytes preparations could be divided into three subpopulations expressing CD16 and/or CD14 in combination with MDC8 ligand (Figure 1B). MDC8 labelling was consistently associated with CD16 expression as described elsewhere [31]. The purity of the pan- and CD16+ monocyte preparations were calculated as 92.7% (IQR: 84–94.2) and 95.02% (IQR: 84.2-95.4), respectively.

**Figure 2:**
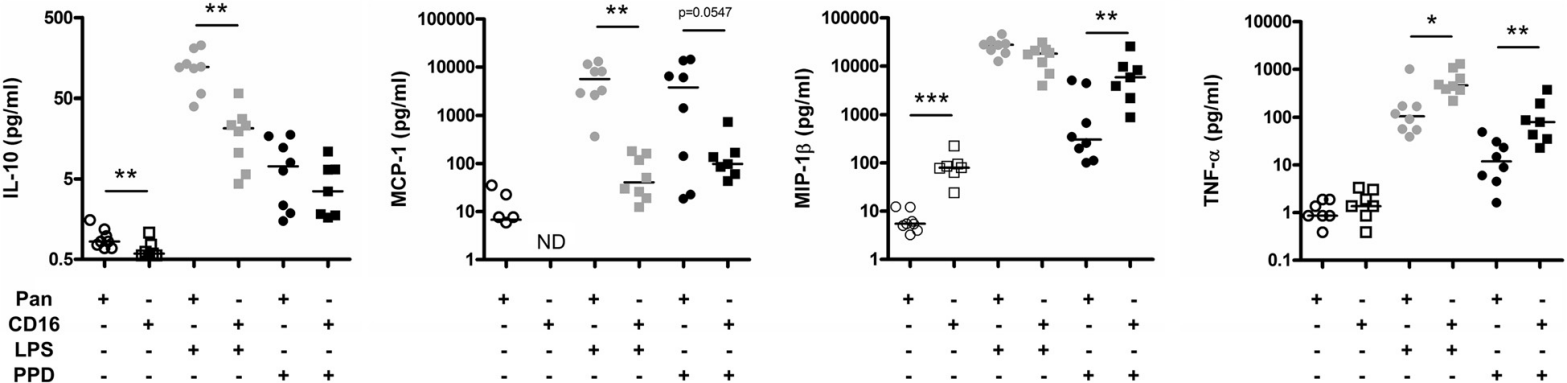
Cytokine and chemokine secretion of pan-monocytes and pro-inflammatory monocytes upon LPS and PPD stimulation. Pan-monocytes (circles) and pro-inflammatory monocyte populations (squares) were subjected to LPS (grey) or *M. tuberculosis* PPD (black) stimulation for 24 h. Supernatants were harvested and analysed for IL-10, MCP-1, MIP-1β and TNF-α levels (ND: not detectable). Data presented as scatter-plots were subjected to Wilcoxon signed rank testing: *p < 0.05, **p < 0.01, *** p < 0.001.

Non-classical or proinflammatory monocytes produce high levels of TNF-α and low concentrations of IL-10 in response to LPS stimulation [32]. We exposed the enriched monocyte preparations to LPS or *M. tuberculosis* derived purified protein derivative (PPD) for 24 h prior harvesting of supernatants and assessment of cytokine and chemokine production (Figure 2). In agreement with published results, CD16^+^ monocyte preparations released significantly more TNF-α after LPS and PPD stimulation and less IL-10 in the context of LPS stimulation when compared to pan-monocytes preparations consisting largely of CD14^+^/CD16^−^ classical monocytes. Similarly, we observed an increased production of Mip1β by CD16+ monocytes after PPD stimulation. No differences between pan- and CD16+ monocyte preparations in their ability to secrete other inflammatory mediators such as IL-6 or IL-1β after LPS and PPD stimulation was observed (data not shown). The secretion of MCP-1 (CCL2) by CD16^+^ monocytes was significantly lower in comparison to autologous pan-monocytes after LPS stimulation (Figure 2). This is in contrast with the observation that CD16^+^ but not CD16^−^ monocytes secrete MCP-1 in response to fractalkine (CX3CL1) receptor ligation [33]. Taken together and since only CD16^−^ circulating monocytes expresses CCR2, the MCP-1 sensor [34], this suggests that while patrolling tissues via CX3CL1-expressing endothelial cells, CD16^+^ can contribute to classical monocyte recruitment through MCP-1 production. However, under PPD stimulation the enhanced secretion of Mip1β which receptor (CCR5) is preferentially expressed by CD16^+^ circulating monocytes suggests that once encountering *M. tuberculosis* infectious foci, CD16^+^ monocytes may only recruit themselves. This interpretation is perfectly consistent with the observation that CD16^+^ monocytes constitute the major monocytic subset infiltrating tuberculous pleural fluid [11].

### Quantitative whole-cell MALDI-TOF MS distinguishes major monocyte subpopulations and captures immune activation events

**Figure 3:**
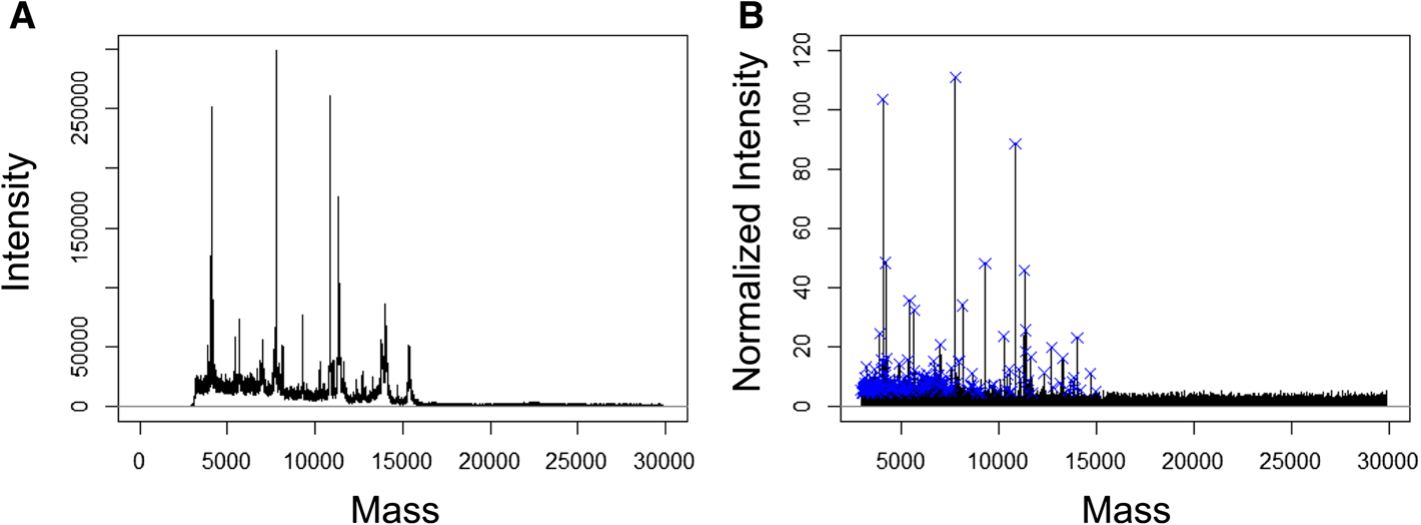
Representative mass spectrum prior and after processing with MALDIquant. A representative mass spectrum from pan-human monocytes prior processing **(A)** and following square-root transformation, smoothing, baseline correction, intensity calibration and spectra alignment is depicted in panel **B**. Blue crosses indicates significant peaks from the peak list obtained after filtering and provided as Additional file 1.

**Figure 4:**
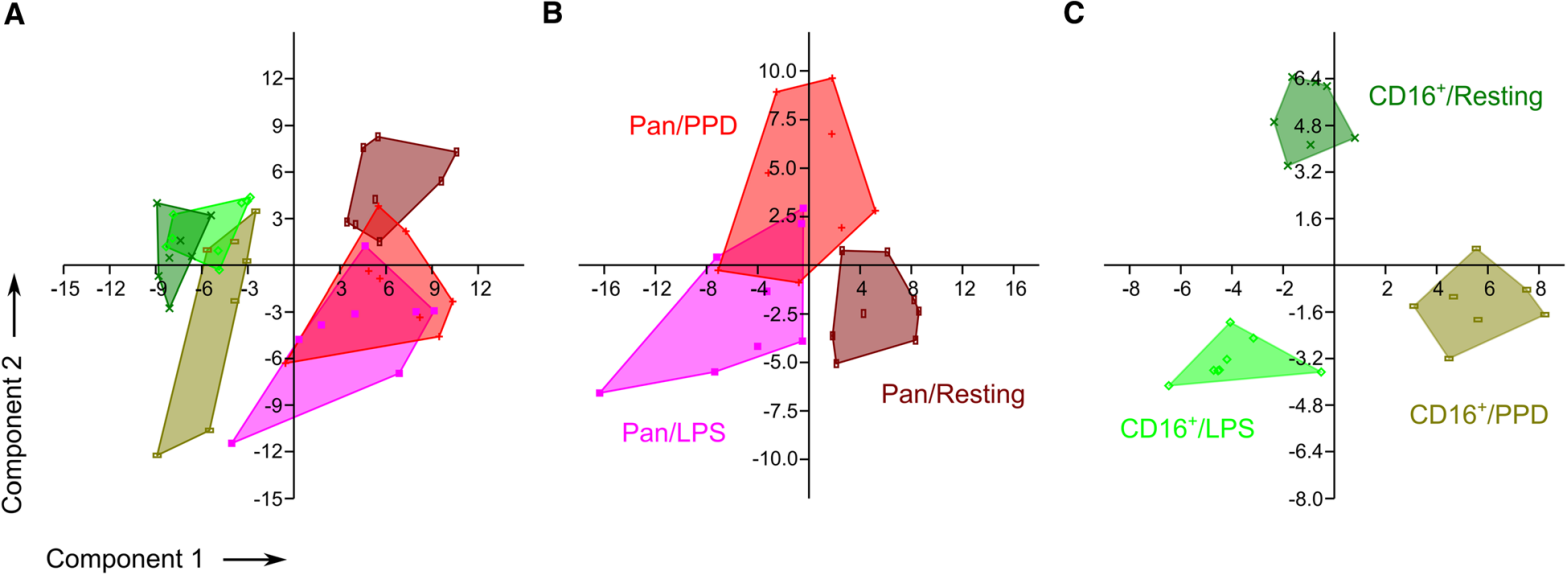
Quantitative whole-cell MALDI-TOF MS discriminates resting and stimulated human monocyte subpopulations. Principal component analysis of all samples combined **(A)**; stimulated and resting pan-monocytes (n = 8) **(B)** and CD16+ monocytes (n = 7) **(C)**, respectively, based on the peak intensity matrix.

Next, we subjected the frozen monocyte cell preparations to whole-cell Matrix Assisted Laser Desorption and Ionization (MALDI) -TOF mass spectrometry analysis. Briefly, cell pellets were lysed, spotted, analysed in quadruplicates. MALDI lacks absolute quantitative power per se. The presence of particular molecular entities may suppress the detection of others for instance. Furthermore, different molecular species have very unequal abilities for desorption and ionisation. Nevertheless, molecular species differing solely from a post-translational modification are likely to behave similarly. Consequently when comparing crude extracts from identical cells cultivated in different conditions, we can assume that variation in intensities or intensity ratios may attest post-translational events or modification of the proteome being analysed. In order to define ratios between different molecular species within and ultimately between different mass spectra, we performed a normalization step so as to set the median intensities of all the peaks detected within one spectrum to one (Figure 3). Mass spectra data were extracted individually to evaluate semi-quantitatively the intensity of m/z peaks with a signal to noise ratio >3 (353 peaks retained). Respective intensities were averaged across technical quadruplicates for statistical analysis. Raw data (e.g. m/z peak list and respective normalized intensities) are presented as Additional file 1. The peak intensity matrix was used for principal component analysis to visualize if individual monocyte preparations with their different stimulation conditions can be separated using whole-cell MALDI-TOF MS. As shown in Figure 4A, PCA component 1 (with 43.4% of the variance) separated unequivocally the pan- from the CD16^+^-monocytes. Interestingly, although pan-monocytes preparations contained a small proportion of CD16^+^ monocytes, our mass spectrometry fingerprinting method identified unambiguously biomarkers from the most abundant cell population. The analyte suppression effect (ASE) [35] or the competition between analytes for ionisation is well acknowledged in MALDI-TOF MS. Ionisation of molecules present in low concentrations is outcompeted by the more abundant ones. We extended the PCA analysis to the m/z peak list intensities specific to the individual pan-monocyte preparations (Figure 4B). Resting pan-monocytes segregated from LPS and PPD stimulated samples whereas the two latter sample groups showed some overlap with each other (Figure 4B). Resting and stimulated CD16+ monocytes segregated unequivocally from each other (Figure 4C).

### Whole-cell MALDI-TOF MS captures molecular patterns of cellular activation specific to monocyte subpopulations.

**Figure 5:**
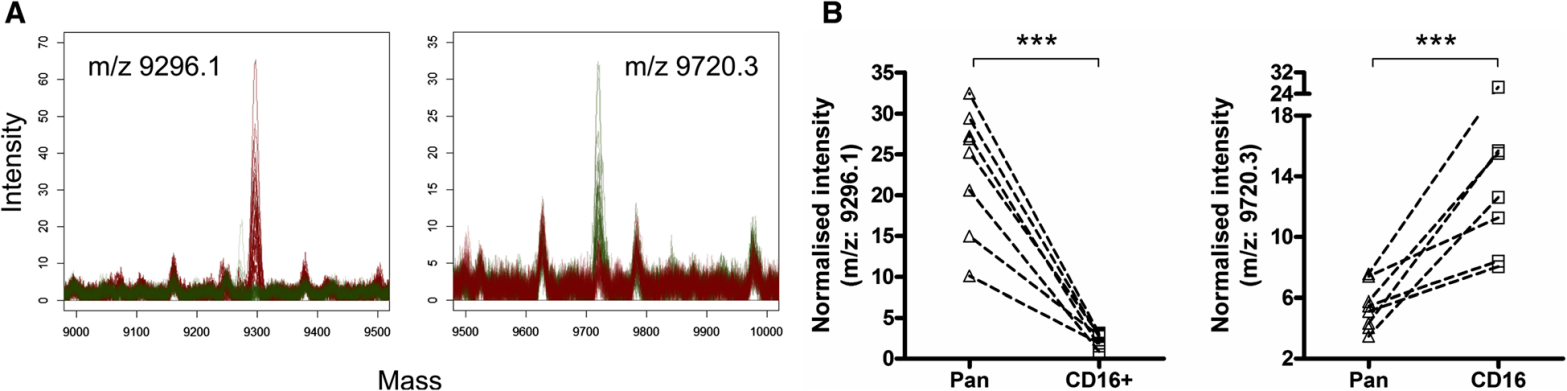
Whole-cell MALDI-TOF MS based identification of biomarkers specific for pan-monocyte and CD16+ monocytes. **A)** Smoothed mass spectra quadruplicates obtained from resting pan-monocytes (n = 8, 32 red spectra) overlaid with those obtained from CD16+ monocytes (n = 7, 28 green spectra). m/z peaks significantly over-represented in pan-monocytes (left overlay plot) or CD16+ monocytes (right overlay plot) are indicated. **B)** Scatter-plot of quadruplicate’s averaged mass peak intensities from pan-monocytes and CD16+ monocytes from individual donor are shown for the peak at 9296.1 m/z (left) and 9720.3 m/z (right). The two-way ANOVA with Bonferroni post-tests correction was used with a significance at p < 0.001.

We subjected the m/z peak intensity list collected to a two-way ANOVA statistical analysis with Bonferroni post-test correction to extract m/z peaks significantly over-represented in the two distinct resting monocyte preparations (pooled data from 7 volunteers). A collection of 25 unique m/z peaks that were significantly associated with either of the pan-monocytes or CD16^+^-monocyte preparations was obtained. In Figure 5, the mass spectra area is given to depict the peaks with the highest expression fold-change (>2) between the two monocyte subpopulations (pan-monocyte markers: m/z 9296.1, 10.24 average fold-change, p < 0.001 and CD16^+^ marker: m/z 9720.3, 2.6 average fold-change, p < 0.001). One would expect that human genetic diversity should lead to quite substantial variability in the mass spectrometry profiles of independent donors. The fact that the relative limited amount of anonymous donors used in this study sufficed to generate significant statistical differences speaks for the robustness of the detected signals. Detection of those masses alone or in combination with other hits could be used as biomarkers for the specific qualitative assessment of the respective monocyte subpopulations.

**Figure 6:**
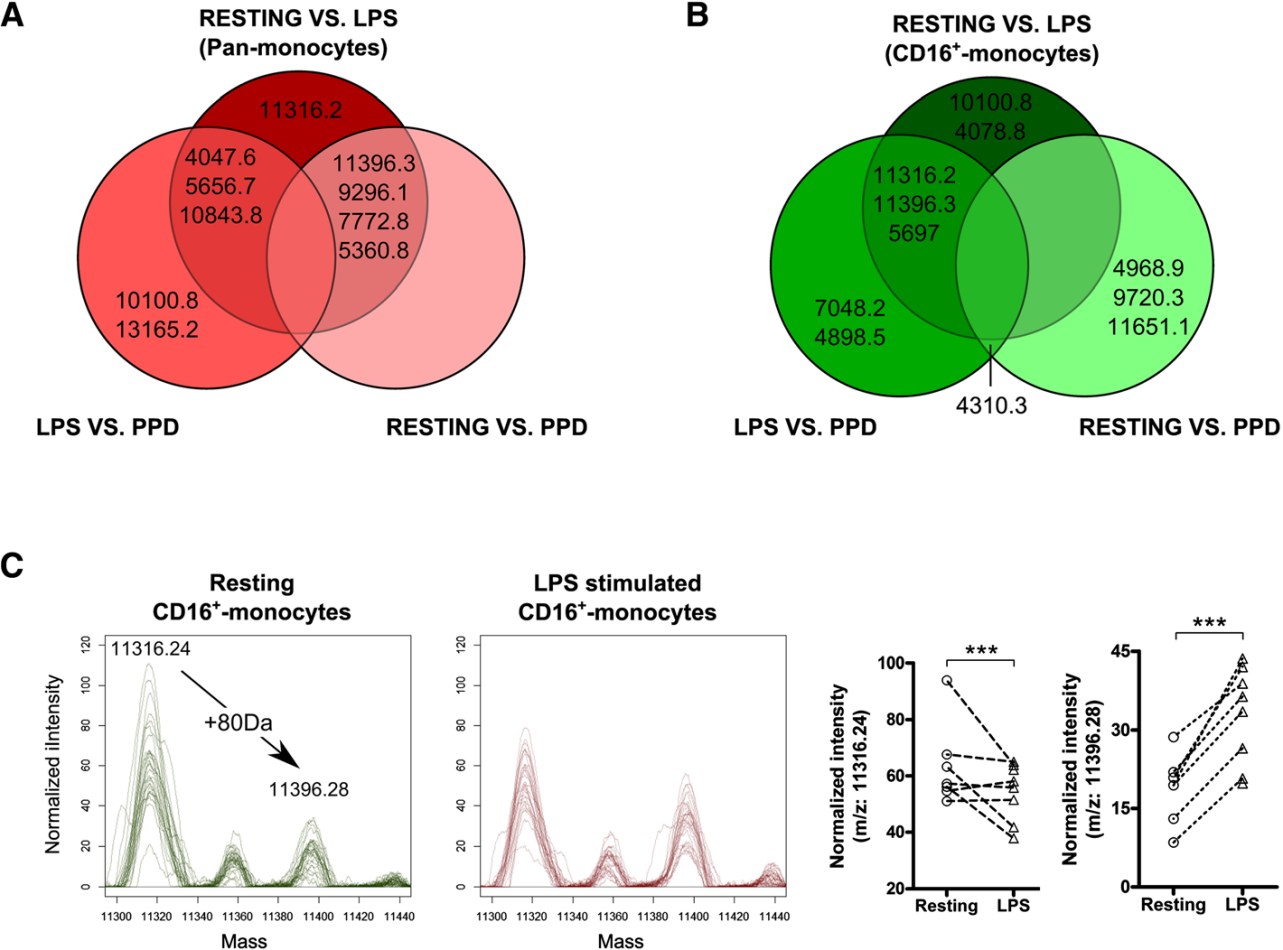
Whole-cell MALDI-TOF can monitor immune activation events. Vent diagrams depicting for **A)** Pan-monocytes or **B)** CD16+ monocytes, m/z peak intensities significantly different between the detailed culture conditions (two-way ANOVA statistical analysis, p < 0.001). **C)** Smoothed mass spectra quadruplicates and respective scatter-plot of quadruplicate’s averaged mass peak intensities from resting CD16^+^ monocytes (green overlay) and LPS-treated CD16^+^ monocytes (red overlay) highlighting m/z areas containing putative phosphorylation of m/z 11316.24 into m/z 11396.28 (two-way ANOVA with Bonferroni post-tests correction, *** p < 0.001).

LPS signals through CD14 via the TLR4-MD-2 pathway [36] whereas PPD triggers the MAPK (ERK1/2/P38) pathway mostly via TLR2 signalling [37]. We next investigated whether whole cell MALDI-TOF MS may offer unbiased insights into immune activation events of monocytes across those two distinct pathways. A two-way ANOVA statistical analysis highlighted respectively 10 and 11 m/z peaks differentially expressed by the pan- and the CD16^+^ monocyte preparations stimulated with LPS or PPD (Figure 6A and B). Three m/z peaks (i.e. 10100.8, 11316.2 and 11396.3) overlapped between pan- and CD16+ monocytes exposed to the two stimuli but their respective pattern behaved remarkably differently. M/z 10100.8 was significantly induced in pan-monocytes exposed to LPS while staying almost unchanged in CD16+ monocytes under the same condition but in turn significantly increased under PPD stimulation conditions. Interestingly, m/z 11316.2 and 11396.3 differ from exactly 80 Da suggesting a potential post-translational modifications e.g. sulfation or phosphorylation [38]. The likelihood of the latter is sustained by the various kinase activities in cell signalling naturally associated to microbial stimulations. Consistent with this hypothesis, we could observe a significant decrease of the precursor at m/z 11316.2 while the putative phosphorylated form increased specifically under LPS stimulation of CD16+ monocytes (Figure 6C). These two peaks were found significantly repressed in pan-monocytes stimulated by either LPS or PPD indicating that the respective pathway would be negatively regulated in stimulated pan-monocytes. Accordingly, within a single spectrum whole-cell mass spectrometry would capture generic activation patterns as well as activation event specific from distinct signalling pathways which remain to be elucidated using for instance, top-down proteomic approaches [39]. After interrogating the human protein SwissProt database using TagIdent (formerly GuessProt) tool and consistent with the blood origin of our biological samples, some relevant candidates may already arise. For instance, the pan-monocyte marker (m/z 9296.1) returned 28 hits including the chemokine CCL13. Similarly, m/z 5697 which was found significantly over-expressed in LPS-treated CD16^+^ monocytes returned 14 hits of which 3 are β − defensins and expected products of an innate immune response upon exposure to a bacterial component. Adequate protein identification involving ms/ms sequencing or peptide mass fingerprints should be performed to confirm the identity of these potential matches. Yet, this analysis brings up that among relatively low-molecular weights species’ candidates stands very relevant protagonists of innate immune responses. In this study, we have systematically performed whole-cell MALDI-TOF on frozen cell pellets which may give rise to criticisms on the potential impact on the freezing step on the subsequent mass spectrometry profiles. In fact, Ouedraogo et al. previously assessed that such freezing procedure does not alter the monocyte fingerprint [40]. However, Munteneanu et al. demonstrated that spectrum richness can be substantially affected by the amount of spotted materials due to the ion suppression effect [28]. Even though, we could readily discriminate monocyte subpopulations and activation, technical improvements may be further achieved by addressing the optimal amount of cells but also the nature of the matrix. Indeed, the profiles obtained in our study using a sinapinic acid matrix differ substantially from monocyte profiles performed with HCCA matrix [27]. Finally, our mass spectrometry profiles were confronted to grouping based on the current monocyte nomenclature and the expression of CD16 and CD14 surface markers [2]. In the future, this classification might be refined based on the differential expression of surface antigens such as Fc gamma receptor I (CD64) [41], P-selectin glycoprotein ligand-1 (PSGL-1) [31], the low-density lipoprotein receptor-related protein (LRP1) [42] or the α2-macroglobulin receptor [43]. This would give rise to new clusters that could be further phenotypically discriminated by whole-cell MALDI-TOF MS profiling.

## Conclusions

To our knowledge, this is the first report describing the use of quantitative whole-cell MALDI-TOF MS analysis to identify mass spectra that discriminate resting and activated human monocyte subsets. As a next step, we are now aiming to identify the most relevant protein candidates highlighted in this study. This proof of concept could easily be translated to clinical studies to monitor the functional status of monocytes from patients suffering from systemic and chronic inflammatory disorders. Diabetes mellitus has been associated with various immune dysfunctions that could be assessed and monitored by whole-cell mass spectrometry approaches. The identification of disease specific markers could provide relevant biological insights in the pathogenesis of diabetes mellitus and the well described enhanced susceptibility of patients to bacterial infection [44]. High throughput monitoring of functional status of cell subsets in peripheral blood based on whole cell MALDI-TOF MS could provide unique opportunities to monitor disease progression and resolution in clinical settings.

## Additional file

Additional file 1:
**MALDIquant processed mass spectrometry data; e.g. m/z peak list and their respective normalized intensities.**

## References

[1] Ginhoux F, Jung S (2014). Monocytes and macrophages: developmental pathways and tissue homeostasis. Nat Rev Immunol.

[2] Ziegler-Heitbrock L, Ancuta P, Crowe S, Dalod M, Grau V, Hart DN (2010). Nomenclature of monocytes and dendritic cells in blood. Blood.

[3] Ziegler-Heitbrock L, Hofer TPJ (2013). Toward a refined definition of monocyte subsets. Front Immunol.

[4] Guilliams M, Ginhoux F, Jakubzick C, Naik SH, Onai N, Schraml BU (2014). Dendritic cells, monocytes and macrophages: a unified nomenclature based on ontogeny. Nat Rev Immunol.

[5] Heimbeck I, Hofer TPJ, Eder C, Wright AK, Frankenberger M, Marei A (2010). Standardized single-platform assay for human monocyte subpopulations: Lower CD14 + CD16++ monocytes in females. Cytom Part J Int Soc Anal Cytol.

[6] Passlick B, Flieger D, Ziegler-Heitbrock HW (1989). Identification and characterization of a novel monocyte subpopulation in human peripheral blood. Blood.

[7] Balboa L, Romero MM, Yokobori N, Schierloh P, Geffner L, Basile JI (2010). Mycobacterium tuberculosis impairs dendritic cell response by altering CD1b, DC-SIGN and MR profile. Immunol Cell Biol.

[8] Hudig D, Hunter KW, Diamond WJ, Redelman D (2014). Properties of human blood monocytes. II. Monocytes from healthy adults are highly heterogeneous within and among individuals. Cytometry B Clin Cytom.

[9] Fingerle G, Pforte A, Passlick B, Blumenstein M, Ströbel M, Ziegler-Heitbrock HW (1993). The novel subset of CD14+/CD16+ blood monocytes is expanded in sepsis patients. Blood.

[10] Thieblemont N, Weiss L, Sadeghi HM, Estcourt C, Haeffner-Cavaillon N (1995). CD14lowCD16high: a cytokine-producing monocyte subset which expands during human immunodeficiency virus infection. Eur J Immunol.

[11] Balboa L, Romero MM, Basile JI, García CA S y, Schierloh P, Yokobori N (2011). Paradoxical role of CD16+CCR2+CCR5+ monocytes in tuberculosis: efficient APC in pleural effusion but also mark disease severity in blood. J Leukoc Biol.

[12] Balboa L, Romero MM, Laborde E, Sabio Y, García CA, Basile JI (2013). Impaired dendritic cell differentiation of CD16-positive monocytes in tuberculosis: role of p38 MAPK. Eur J Immunol.

[13] Frankenberger M, Ekici AB, Angstwurm MW, Hoffmann H, Hofer TPJ, Heimbeck I (2013). A defect of CD16-positive monocytes can occur without disease. Immunobiology.

[14] Ghattas A, Griffiths HR, Devitt A, Lip GYH, Shantsila E (2013). Monocytes in coronary artery disease and atherosclerosis: where are we now?. J Am Coll Cardiol.

[15] Chiba T, Umegaki K (2013). Pivotal roles of monocytes/macrophages in stroke. Mediators Inflamm.

[16] Kawanaka N, Yamamura M, Aita T, Morita Y, Okamoto A, Kawashima M (2002). CD14+, CD16+ blood monocytes and joint inflammation in rheumatoid arthritis. Arthritis Rheum.

[17] Min D, Brooks B, Wong J, Salomon R, Bao W, Harrisberg B (2012). Alterations in monocyte CD16 in association with diabetes complications. Mediators Inflamm.

[18] Naert G, Rivest S (2013). A deficiency in CCR2+ monocytes: the hidden side of Alzheimer’s disease. J Mol Cell Biol.

[19] Yamasaki R, Lu H, Butovsky O, Ohno N, Rietsch AM, Cialic R (2014). Differential roles of microglia and monocytes in the inflamed central nervous system. J Exp Med.

[20] Karlmark KR, Tacke F, Dunay IR (2012). Monocytes in health and disease - Minireview. Eur J Microbiol Immunol.

[21] Gate D, Rezai-Zadeh K, Jodry D, Rentsendorj A, Town T (2010). Macrophages in Alzheimer’s disease: the blood-borne identity. J Neural Transm Vienna Austria 1996.

[22] Giebel R, Worden C, Rust SM, Kleinheinz GT, Robbins M, Sandrin TR (2010). Microbial Fingerprinting using Matrix-Assisted Laser Desorption Ionization Time-Of-Flight Mass Spectrometry (MALDI-TOF MS). In Advances in Applied Microbiology.

[23] Zhang X, Scalf M, Berggren TW, Westphall MS, Smith LM (2006). Identification of mammalian cell lines using MALDI-TOF and LC-ESI-MS/MS mass spectrometry. J Am Soc Mass Spectrom.

[24] Vogel G, Strauss A, Jenni B, Ziegler D, Dumermuth E, Antz S (2011). Development and validation of a protocol for cell line identification by MALDI-TOF MS. BMC Proc.

[25] Hanrieder J, Wicher G, Bergquist J, Andersson M, Fex-Svenningsen Å (2011). MALDI mass spectrometry based molecular phenotyping of CNS glial cells for prediction in mammalian brain tissue. Anal Bioanal Chem.

[26] Povey JF, O’Malley CJ, Root T, Martin EB, Montague GA, Feary M (2014). Rapid high-throughput characterisation, classification and selection of recombinant mammalian cell line phenotypes using intact cell MALDI-ToF mass spectrometry fingerprinting and PLS-DA modelling. J Biotechnol.

[27] Ouedraogo R, Daumas A, Ghigo E, Capo C, Mege J-L, Textoris J (2012). Whole-cell MALDI-TOF MS: A new tool to assess the multifaceted activation of macrophages. J Proteomics.

[28] Munteanu B, von Reitzenstein C, Hänsch GM, Meyer B, Hopf C (2012). Sensitive, robust and automated protein analysis of cell differentiation and of primary human blood cells by intact cell MALDI mass spectrometry biotyping. Anal Bioanal Chem.

[29] Gibb S, Strimmer K (2012). MALDIquant: a versatile R package for the analysis of mass spectrometry data. Bioinforma Oxf Engl.

[30] Hammer O, Harper DAT, Ryan PD (2001). PAST: Paleontological statistics software package for education and data analysis. Palaeontologia Electronica.

[31] De Baey A, Mende I, Riethmueller G, Baeuerle PA (2001). Phenotype and function of human dendritic cells derived from M-DC8(+) monocytes. Eur J Immunol.

[32] Frankenberger M, Sternsdorf T, Pechumer H, Pforte A, Ziegler-Heitbrock HW (1996). Differential cytokine expression in human blood monocyte subpopulations: a polymerase chain reaction analysis. Blood.

[33] Ancuta P, Wang J, Gabuzda D (2006). CD16+ monocytes produce IL-6, CCL2, and matrix metalloproteinase-9 upon interaction with CX3CL1-expressing endothelial cells. J Leukoc Biol.

[34] Weber C, Belge KU, von Hundelshausen P, Draude G, Steppich B, Mack M (2000). Differential chemokine receptor expression and function in human monocyte subpopulations. J Leukoc Biol.

[35] Kamp RM, Calvete JJ, Choli-Papadopoulou T (2004). Methods in Proteome and Protein Analysis.

[36] Park BS, Lee J-O (2013). Recognition of lipopolysaccharide pattern by TLR4 complexes. Exp Mol Med.

[37] Yang C-S, Shin D-M, Lee H-M, Son JW, Lee SJ, Akira S (2008). ASK1-p38 MAPK-p47phox activation is essential for inflammatory responses during tuberculosis via TLR2-ROS signalling. Cell Microbiol.

[38] Parker CE, Mocanu V, Mocanu M, Dicheva N, Warren MR, Alzate O (2010). Mass Spectrometry for Post-Translational Modifications. Neuroproteomics.

[39] Catherman AD, Skinner OS, Kelleher NL (2014). Top Down proteomics: facts and perspectives. Biochem Biophys Res Commun.

[40] Ouedraogo R, Flaudrops C, Ben Amara A, Capo C, Raoult D, Mege J-L (2010). Global analysis of circulating immune cells by matrix-assisted laser desorption ionization time-of-flight mass spectrometry. PLoS One.

[41] Grage-Griebenow E, Lorenzen D, Fetting R, Flad HD, Ernst M (1993). Phenotypical and functional characterization of Fc gamma receptor I (CD64)-negative monocytes, a minor human monocyte subpopulation with high accessory and antiviral activity. Eur J Immunol.

[42] Ferrer DG, Jaldín-Fincati JR, Amigone JL, Capra RH, Collino CJ, Albertini RA (2014). Standardized flow cytometry assay for identification of human monocytic heterogeneity and LRP1 expression in monocyte subpopulations: decreased expression of this receptor in nonclassical monocytes. Cytom Part J Int Soc Anal Cytol.

[43] Hudig D, Hunter KW, Diamond WJ, Redelman D (2014). Properties of human blood monocytes. I. CD91 expression and log orthogonal light scatter provide a robust method to identify monocytes that is more accurate than CD14 expression. Cytometry B Clin Cytom.

[44] Hodgson K, Morris J, Bridson T, Govan B, Rush C, Ketheesan N (2015). Immunological mechanisms contributing to the double burden of diabetes and intracellular bacterial infections. Immunology.

